# Local Stabilization of Hypoxia-Inducible Factor-1α Controls Intestinal Inflammation *via* Enhanced Gut Barrier Function and Immune Regulation

**DOI:** 10.3389/fimmu.2020.609689

**Published:** 2021-01-14

**Authors:** Young-In Kim, Eun-Je Yi, Young-Dae Kim, A Reum Lee, Jiwoung Chung, Hae Chan Ha, Joong Myung Cho, Seong-Ryeol Kim, Hyun-Jeong Ko, Jae-Hee Cheon, Yong Rae Hong, Sun-Young Chang

**Affiliations:** ^1^Laboratory of Microbiology, College of Pharmacy and Research Institute of Pharmaceutical Science and Technology, Ajou University, Suwon, South Korea; ^2^Institute for Drug Discovery, CrystalGenomics, Inc., Seongnam-si, South Korea; ^3^Laboratory of Microbiology and Immunology, Department of Pharmacy, Kangwon National University, Chuncheon-si, South Korea; ^4^Department of Internal Medicine and Institute of Gastroenterology, Yonsei University College of Medicine, Seoul, South Korea

**Keywords:** inflammatory bowel disease, gut barrier, hypoxia-inducible factor, prolyl hydroxylase inhibitor, immune regulation

## Abstract

Intestinal epithelial cells are adapted in mucosal hypoxia and hypoxia-inducible factors in these cells can fortify barrier integrity to support mucosal tissue healing. Here we investigated whether hypoxia-related pathways could be proposed as potential therapeutic targets for inflammatory bowel disease. We developed a novel hypoxia-inducible factor (HIF) prolyl hydroxylase inhibitor, CG-598 which stabilized HIF-1α in the gut tissue. Treatment of CG-598 did not affect extra-intestinal organs or cause any significant adverse effects such as erythropoiesis. In the experimental murine colitis model, CG-598 ameliorated intestinal inflammation with reduction of inflammatory lesions and pro-inflammatory cytokines. CG-598 treatment fortified barrier function by increasing the expression of intestinal trefoil factor, CD73, E-cadherin and mucin. Also, IL-10 and IL-22 were induced from lamina propria CD4^+^ T-cells. The effectiveness of CG-598 was comparable to other immunosuppressive therapeutics such as TNF-blockers or JAK inhibitors. These results suggest that CG-598 could be a promising therapeutic candidate to treat inflammatory bowel disease.

## Introduction

The intestine harbor huge numbers of immunocompetent cells to manage elaborate interactions with microbes, for tolerance to luminal microbiota, as well as protection against invading pathogens. When genetic susceptibility is combined with environmental factors, chronic uncontrolled inflammatory diseases such as inflammatory bowel disease (IBD) are initiated (1). Inflammatory bowel diseases, including Crohn’s disease and ulcerative colitis, are clinically chronic relapsing diseases associated with dysregulated microbial composition (dysbiosis) and defective immune regulation. To investigate IBD therapeutics, various approaches have mainly focused on control of inflammation using anti-inflammatory drugs or immunosuppressants because inflammation is the obvious and most important final disease outcome regardless of the complicated pathogenesis of IBD (2). Recently, biologics for blockade of tumor necrosis factor (TNF) or integrin, key mediators of pathogenic inflammation have been increasingly applied. In addition, clinical therapies as blockades of cytokine signaling *via* inhibition of intracellular Janus kinases (JAKs) such as tofacitinib were developed. However, novel fundamental modulators from an understanding of underlying pathology should be investigated.

The intestinal epithelium provides a physical and innate defense barrier against noxious luminal triggers (3). The immune cells in the lamina propria can initiate inflammatory responses against invaders if these barriers are injured. Unlike the skin barrier, the intestinal epithelium has a monolayer structure and acts as a physical barrier as well as a coordinating hub to manage dynamic luminal conditions. To efficiently absorb nutrients digested from food, the epithelium of the small intestine has thin and permeable layers spread over a vast surface area. During infection with bacterial pathogens, epithelial sheets are easily shed from the basement membranes of tissue with accompanying host cell death (4). To recover the intestinal barrier following infection, rapid production of epithelial cells from intestinal stem cells are stimulated by endogenous signals and commensal-derived short chain fatty acid (3). Defects in the gut barrier are associated with a broad range of human intestinal diseases as well as extra-intestinal diseases such as non-alcoholic fatty liver disease and neurologic brain disease (5).

In the physiology of the intestine, the concentration of oxygen varies from the highly vascularized lamina propria to the anaerobic lumen. The estimated oxygen partial pressure (pO_2_) is <15 mmHg in the lumen, 23 mmHg at the tip of villi, and 128–160 mmHg in the blood vessels of submucosa (6–8). Consistent with other mammalian cells, the intestinal epithelial cells utilize hypoxia-induced mechanisms to respond to low oxygen conditions. Hypoxia responses are modulated and adapted by oxygen-sensitive transcription factors such as hypoxia-inducible factor (HIF). These inducible factors consist of an oxygen-sensitive α-subunit (HIF-1α, HIF-2α, and HIF-3α) and a conserved β-subunit, HIF-1β, also known as aryl hydrocarbon receptor nuclear translocator (9). Under normoxia, prolyl hydroxylases (PHDs) and asparaginyl hydroxylase factor inhibiting HIF (FIH) hydroxylate the HIF-α subunit at proline or asparagine residues leading to their proteasomal degradation or inhibition of their interaction with a cofactor CREB binding protein (CBP). However, hypoxia-induced inhibition of PHD and FIH stabilizes HIF-α. After translocation of the HIF-α subunit to the nucleus, it complexes with HIF-β to form an active transcription factor HIF, which binds to hypoxia responsive elements (HREs) in the promoter regions of target genes followed by initiation of transcription (10).

Hypoxia-induced signaling by HIFs can promote or counteract mucosal inflammatory responses depending on the cell types. Hypoxia induces inflammation in the intestine and may represent an environmental cause for IBD pathogenesis. In fact, both HIF-1α and HIF-2α are found at high levels in intestinal epithelial cells (IEC) from patients with active ulcerative colitis or Crohn’s disease (11). Hypoxia stimulates IECs to produce TNF which increases barrier permeability (12). Under hypoxic conditions, innate immune cells including neutrophils, macrophages, and dendritic cells resist apoptosis and produce more pro-inflammatory cytokines (13–15). Otherwise, hypoxia-exposed IEC at physiological conditions promote barrier-preservative functions to reduce the inflammatory burden by up-regulating intestinal trefoil factor (ITF), mucin-3, and CD73. The involvement of HIFs in IBD pathogenesis has been investigated in experimental animal models. The HIF-1α isoform is beneficial to repression of oxazolone- and 2,4,6-trinitrobenzene sulfonic acid (TNBS)-induced colitis *via* induction of barrier-protective genes whereas HIF-2α is detrimental to dextran sulfate sodium (DSS)-induced colitis *via* increased inflammatory responses (16, 17). These results suggest differential effects of HIF isoforms in gut homeostasis.

The PHDs consist of three isoforms, PHD1, PHD2, and PHD3, which control diverse functions in immune and non-immune cells. In contrast, little information about the cell-type specific effects of FIH has been accumulated. A defect of PHD1 or PHD3 exhibits protective effects on intestinal epithelial barrier integrity in mice (18, 19). The protective role of HIF-1α and PHD blockade during gut inflammation led to the investigation of hydroxylase inhibitors as a potential therapeutic strategy. The pan-hydroxylase inhibitors such as dimethyloxalylglycine (DMOG) and FG-4497 have protective effects in experimental murine colitis (20, 21). A candidate of a class of HIF-1α–selective PHD inhibitors, AKB-4924, exhibits protective effects in TNBS-induced colitis (22). Oral administration of AKB-4924 reduced systemic off-target effects in extra-intestinal organs while maintaining the localized beneficial effect for colonic inflammation (23). Similarly, oral administration of the PHD inhibitor, TRC160334, showed therapeutic effects in chemically induced murine colitis (24). Several hydroxylase inhibitors are currently under investigation for the treatment of various diseases such as inflammatory bowel disease (23), chronic kidney disease-related anemia (25), and cancer (26). However, long-term systemic treatment with pan-hydroxylase inhibitors has raised the possibility of promotion of cancer, erythropoiesis, and fibrosis in the kidney or liver, and altered biochemical pathways.

Here, we synthesized CG-598, a novel HIF-1α stabilizer *via* inhibition of PHD. At the early stage of our study, CG-598 was a candidate for anemia therapeutics. However, because CG-598 can be locally distributed in the gut after oral administration without significant systemic absorption which may cause unwanted adverse effects, we applied it to IBD therapeutics. Therefore, we investigated whether CG-598 has beneficial effects in colitis and may be a novel candidate for IBD therapeutics which selectively target the gut.

## Materials and Methods

### HIF-PHD Inhibitor CG-598

**Figure 1:**
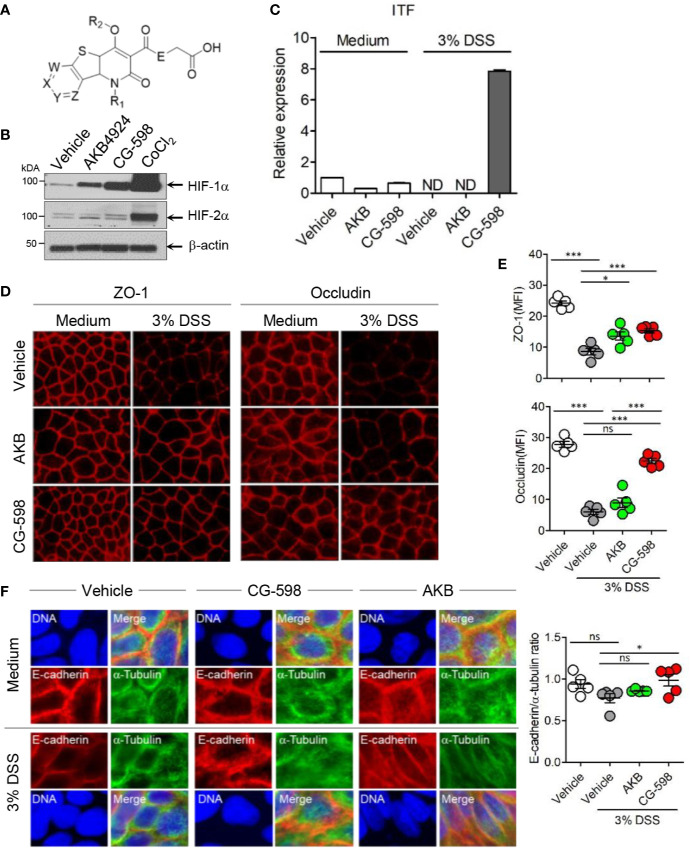
The HIF-1α stabilizer CG-598 enhances barrier-associated molecules in intestinal epithelial cells *in vitro*. **(A)** The chemical structure of CG-598. **(B)** HeLa cells were treated with 10 μM AKB-4924, CG-598, or CoCl_2_ for 24 h. Whole cell extracts from each treatment group were analyzed to detect HIF-1α and HIF-1β. **(C)** HCT116 cells were pre-treated with 10 μM AKB-4924 or CG-598 for 2 h and further cultured with 3% DSS for 24 h. The *itf* mRNA level was measured by real-time PCR. **(D–F)** Caco-2 cells were pre-treated with 10 μM AKB-4924 or CG-598 for 1 h and further incubated with 3% DSS for 24 h. Mean fluorescence intensity (MFI) or ratio was summarized. **(D, E)** Immunofluorescence of ZO-1, occludin, **(F)** E-cadherin (red) and α-tubulin (green). One-way ANOVA. Data are shown as mean ± SEM, ns, not significant, *p < 0.05, ***p < 0.001.

CG-598 (C_21_H_17_N_3_O_5_S, MW 423.4) is a novel HIF-PHD inhibitor which was designed and synthesized by CrystalGenomics, Inc (**Figure 1A**). The full structure is disclosed in Korean patent 10-2019-0112154. The values of the partition coefficient (clogP) and polar surface area for CG-598 were 1.36 and 148.07, respectively. To assess the biological activity of CG-598, a prolyl hydroxylation reaction was measured. A peptide (FITC-Asp-Asp-Leu-Asp-Leu-Glu-Ala-Leu-Ala-Pro-Tyr-Ile-Pro-Ala-Asp-Asp-Asp-Phe-Gln-Leu-Arg-OH) was synthesized by GL Biochem Ltd. (Shanghai, China). The immobilized peptide substrate was incubated with the concentration range from 0.003 to 100 μM of CG-598 in 20 mM Tris-HCl, pH 7.5, 100 mM NaCl, 0.1 mM α-ketoglutarate, 2 mM ascorbic acid, 100 μM FeCl_2_, 0.5% nonidet P40 in a final volume of 25 μL at 30°C for 60 min. To stop the reaction, plates were incubated at 95°C for 1 min and was cooled at 0°C for 5 min. Hydroxylated HIF-1α was incubated with 125 nM glutathione S-transferase-tagged VHL/Elongin B/Elongin C in 25 μL binding buffer (50 mM Tris-HCl, pH 7.5, 120 mM NaCl, 0.5% nonidet P40) for 30 min at room temperature. The amount of bound VBC was determined by measuring the fluorescence polarization with a plate reader (Molecular Devices). All pan-PHD inhibitors including PN-3602/compound16 (27), AKB-4924 (28), and CG-690, candidates for anemia therapeutics, were previously reported or synthesized by CrystalGenomics, Inc.

### Western Blotting

HeLa cells (ATCC, Manassas, VA, USA) were cultured in Minimum Essential Medium (MEM) supplemented with 10% fetal bovine serum (FBS; GIBCO-BRL, NY, USA). HeLa cells seeded at a density of 1.5 × 10^6^ cells/well in 60 mm culture dishes were treated with 10 µM CG-598, 10 µM AKB-4924, or 500 µM CoCl_2_ (Sigma-Aldrich, St. Louis, MO, USA) for 24 h. Whole cell lysates were prepared using radioimmunoprecipitation assay (RIPA) buffer. To prepare nuclear extracts, murine colons were homogenized in buffer A (10 mM HEPES, 10 mM KCl, 0.1 mM EDTA, 1 mM 1,4-dithiothreitol (DTT), and 10% NP-40, with protease inhibitors) and incubated 15 min on ice. An additional aliquot of 10% NP-40 was added, and samples were centrifuged. The supernatant was discarded and buffer B (20 mM HEPES, 0.4 M NaCl, 1 mM EDTA, 1 mM DTT, with protease inhibitors) was added to the pellet and incubated at 4°C for 1 h. After centrifugation, the protein concentration of each pellet was quantified. To prepare cytosolic colon extracts, tissues were homogenized in pro-prep protein extraction solution (iNtRON Biotechnology, Seongnam, Korea) with protease inhibitors, then placed at −20°C overnight. Homogenates were centrifuged, supernatants discarded and protein concentrations of pellets were quantified. Western blotting was performed to investigate the protein expression of HIF-1α and HIF-2α. The following antibodies were used: anti-β-actin (Santa Cruz Biotechnology, CA, USA), anti-lamin B1(Bioworld Technology, MN, USA), anti-HIF-1α (BD Biosciences Pharmingen, CA, USA for HeLa cells; Novus Biologicals, CO, USA for colon tissues), anti-HIF-2α (Novus Biologicals, CO, USA), horseradish peroxidase (HRP)-conjugated anti-rabbit IgG, and HRP-conjugated anti-mouse IgG (Santa Cruz Biotechnology). Visualization of protein bands was accomplished using an electrochemiluminescence kit (Advansta, CA, USA). Representative results from at least three independent experiments are shown.

### Gene Expression

For *in vitro* gene expression experiments, HCT116 cells which originated from colorectal carcinoma (ATCC) were pre-treated with 10 μM AKB-4924 or 10 μM CG-598 for 2 h and further incubated with 3% DSS for 24 h. For *in vivo* gene expression, colon tissues from mouse experiments were isolated, washed, and homogenized. Total RNA from the cell lines or tissue homogenates were extracted using TRIzol reagent (Invitrogen) according to the manufacturer’s instructions. cDNA was synthesized using SuperScript IV First-Strand Synthesis System (Thermo Fisher Scientific, MA, USA), following the manufacturer’s instructions. SYBR Green™ Premix Ex TaqTM II Kit (Takara, RR820B) was used for performing the RT-qPCR reaction. Amplification was performed with CFX Connect Real-Time PCR system (BioRad) under the following conditions: 95°C for 30 s of pre-denaturation, denaturation at 95°C for 5 s, and annealing at 62°C for 30 s for 40 cycles. Primers used for PCR were as follows: ITF F 5′-ATGGCTGCCAGAGCGCTCTGCAT-3′, R 5′-TGCCTCAGAAGGTGCATTCTGCT-3′, MUC2 F 5′-GCTGACGAGTGGTTGGTGAATG-3′, R 5′-GATGAGGTGGCAGACAGGAGAC-3′, MUC3 F 5′-AACTGCAGCTACGGCAAATGTC-3′, R 5′-AGGTTTCGCCTACCA TCGTA AC-3′, IL-22 F 5′-TTGAGGTGTCCAACTTCCAGCA-3′, R 5′-AGCCGGACGTCTGTGTTGTTA-3′, E-cadherin F 5′-CAGCCTTCTTTTCGGAAGACT-3′, R 5′-GGTAGACAGCTCCCTATGACTG-3′, IL-10 F 5′-GCC ACATGCTCCTAGAGCTG-3′, R 5′-CAGCTGGTCCTTTGTTTGAAA-3′, β-actin F 5′-TAGGCGGACTGTTACTGAGC-3′, R 5′-TGCTCCAACCAACTGCTGTC-3′, GAPDH F 5′-AACTTTGGGATTGTGGAAGG-3′, R 5′-ACACATTGGGGGTAGGAACA-3′ (as a housekeeping gene and protein normalization). Each sample was run in triplicate and a minimum of two independent experiments were performed for each sample.

### Immunofluorescence Imaging

Caco-2 cells (KCLB, Seoul, Korea) were cultured in MEM medium supplemented with 20% FBS. For imaging of occludin and tight junction protein ZO-1, Caco-2 cells were seeded on coverslips at 1.5 × 10^5^ cells/well in 12-well cell culture plates and pre-treated with 10 µM CG-598 or AKB-4924 for 1 h and further incubated with 3% DSS for 24 h. After fixation with methanol: acetone (1:1) for 5 min at −20°C and blocking with 5% bovine serum albumin (BSA)/phosphate buffered saline (PBS) for 30 min, the cells were stained overnight at 4°C with anti-occludin or anti-ZO-1 followed by incubation with Alexa Fluor 594 conjugated donkey anti-rabbit IgG (H+L) Ab (all antibodies obtained from Invitrogen) for 1 h at room temperature. For E-cadherin imaging, HCT116 cells were seeded on coverslips at 2 × 10^5^ cells/well within 12-well cell culture plates and pre-treated with 10 µM CG-598 or AKB-4924 for 1 h and further incubated with 3% DSS for 24 h. After fixation with 4% formaldehyde for 20 min at room temperature and blocking in 5% BSA in PBS for 30 min, cells were stained overnight at 4°C with anti-α-tubulin (clone DM1A; Santa Cruz Biotechnology) and anti-E-cadherin (clone 24E10; Cell Signaling Technology, MA, USA) followed by Alexa Fluor 488 conjugated donkey anti-mouse IgG (H+L) or Alexa Fluor 594 conjugated donkey anti-rabbit IgG (H+L) Ab (both from Invitrogen), respectively. Slides were mounted with fluorescence mounting medium (Dako, CA, USA) and visualized with the DMi8 fluorescent microscope (Leica Microsystems). The mean fluorescence intensity was analyzed by ImageJ (National Institutes of Health). For E-cadherin imaging of intestinal epithelium, colon tissues were fixed in 4% formaldehyde and embedded in paraffin. The 5-µm sliced tissues were deparaffinized and then incubated 121°C for 10 min in Retrievagen A (BD Pharmingen, CA, USA). After blocking with 10% FBS in PBS for 30 min, slides were stained overnight at 4°C with Alexa Fluor 488 conjugated anti-E-cadherin (clone 36/E-Cadherin; BD Pharmingen) and mounted with ProLong Gold Antifade Reagent with 4′,6-diamidino-2-phenylindole (DAPI; Invitrogen). Slides were visualized with an A1R HD25 N-SIM S confocal microscope (Nikon).

### Animals

All animal experiments except DSS-induced colitis experiments were approved by the Institutional Animal Care and Use Committee of Crystal Genomics (CG-IACUC-19017). DSS-induced colitis experiments were approved by the Institutional Animal Care and Use Committee of Ajou University (IACUC No. 2017-0021). Animals were kept in the Laboratory Animal Research Center of Ajou University Medical Center under specific pathogen-free conditions or in the animal center of Crystal Genomics following institutional guidelines and received sterilized food and water *ad libitum*. Five-week-old female wild-type (WT) C57BL/6 mice and male SD rats were purchased from Orient Bio Inc. (Sungnam, Korea).

### Tissue Distribution of CG-598 and Hematology

To investigate the absorption of CG-598 into intestine or systemic blood, C57BL/6 mice (n=3 per group) were orally administered with 150 mg/kg of CG-598 in 1% carboxymethyl cellulose (CMC). Colon tissues and plasma were sampled at 1, 2, 4, and 8 h following oral administration of CG-598. To analyze the excretion of CG-598, fasted male SD rats (n=3) were orally administered with 150 mg/kg of CG-598. After dosing, feces and urine were collected separately during 0 to 4 h, 4 to 8 h, 8 to 24 h, and 24 to 48 h and the content of CG-598 was quantified by measuring with Micromass/HPLC Alliance 2795 and analyzing with QuantiLynx (Waters, MA, USA). To analyze generation of reticulocytes/red blood cells from bone marrow, male ICR mice (n=5 per group) were orally fed once a day with the indicated doses of CG-598 or 100 mg/kg PN-3602. Body weights were monitored every 4 days. At 15 days after the first oral administration, blood samples were analyzed *via* auto hematology analyzer (BC-2800vet, Mindary).

### Colitis

For dinitrobenzene sulfonic acid (DNBS)-induced colitis experiments, C57BL/6 mice were administered intra-rectally with 3% DNBS (Sigma Aldrich) resuspended in 50 μL of 30% ethanol in PBS (n=9 or 10 per group). On day 4, mice were examined histologically, and colon tissue lengths were measured. In a murine model of DSS-induced colitis, 2.5% DSS (molecular weight 36,000–50,000 Da; MP Biomedicals LLC) dissolved in autoclaved drinking water was provided *ad libitum* to C57BL/6 mice for five days and then exchanged with normal drinking water until the end of the experiment on day 12. Mice were treated with CG-598 or AKB-4924 in 1% CMC (Sigma Aldrich)/0.1% Tween 80 by oral gavage once every day starting from DSS treatment. AKB-4924 was described previously to be prepared in 40% 2-hydroxylpropyl-beta-cyclodextrin in 50 mM aqueous citrate buffer at pH 4 (28), however, 1% CMC was used to have an identical vehicle with CG-598. Mice were treated intraperitoneally with 0.5 mg anti-mouse TNF-α antibody (Bio X Cell) on days 4, 6, 8, and 10. During the entire experiment, body weights of mice and disease activity index including rectal bleeding and diarrhea were monitored daily as described previously (29). On day 11 or 12, gross lengths of colon were measured, and colons were examined histologically. To determine bacterial translocation, the spleen and mesenteric lymph nodes (MLN) isolated from mice at day 11 were homogenized and serial dilutions were plated on Luria broth agar plates and incubated 37°C overnight. Colony-forming units (CFU)/g were determined.

### Histology

Colon tissues fixed in neutral-buffered formalin were trimmed, processed, and embedded in paraffin. H&E-stained colonic tissue sections were scored by a gastrointestinal pathologist (Asan medical center) as a blinded manner to the experimental groups and timing according to the following semi-quantitative measurement system: severity of inflammation (0, rare inflammatory cells in the lamina propria; 1, increased numbers of granulocytes in the lamina propria; 2, confluence of inflammatory cells extending into the submucosa; and 3, transmural extension of the inflammatory infiltrate), crypt damage (0, intact crypts; 1, loss of one-third basal crypts; 2, loss of two-thirds basal crypts; 3, entire crypt loss; 4, change of epithelial surface with erosion; and 5, confluent erosion), ulceration (0, absence of ulcers; 1, one or two foci of ulcerations; 2, three or four foci of ulcerations; and 3, confluent or extensive ulceration). Values from three parameters were added to give a maximal histological score of 11 (30).

### Cytokine and Treg Analysis

Whole colon tissues were homogenized using a tissue homogenizer (Minilys personal homogenizer, Bertin). Tissue homogenates were analyzed for pro- and anti-inflammatory cytokines. TNF-α, IFN-γ, IL-6, MCP-1, and IL-10 were analyzed by flow cytometry using BD Cytometric Beads Assay Mouse inflammation kit (BD Biosciences). IL-1β and IL-22 were detected using mouse IL-1β and IL-22 ELISA kits (Invitrogen) following the manufacturer’s instructions. To isolate CD4^+^ T-cells, colons were inverted on polyethylene tubes (Becton Dickinson, Franklin Lakes, NJ, USA) and washed with PBS three times. Colons were further treated with 1 mM DTT (DTT; Sigma-Aldrich) and 30 mM EDTA to remove mucus and epithelium, respectively and were digested with 108 U/mL type IV collagenase (Sigma-Aldrich) for 90 min at 37°C. Isolated cells were treated using a discontinuous density gradient containing 66% and 44% Percoll (GE Healthcare Life Sciences, Uppsala, Sweden). CD4^+^ T-cells and CD8^+^ T-cells were sorted using BD FACSAria III (> 95% purity). To analyze IL-10- and IL-22-secreting T-cells, isolated cells were stimulated on plates coated with anti-CD3/anti-CD28 (Invitrogen) overnight. Culture supernatant was collected for cytokine analysis. For analysis of CD4^+^ Treg, cells were stained with anti-mouse CD4 (clone RM4-5; Invitrogen) followed by staining for Helios (clone 22F6; Invitrogen) and Foxp3 (clone FJK-16s; Invitrogen) after intracellular fixation and analyzed by flow cytometry (BD FACSAria III).

### Statistical Analysis

Student’s *t*-test was used to compare differences between two groups. To compare multiple groups, we performed a one-way or two-way analysis of variance (ANOVA) followed by Tukey’s multiple comparison test. Values of *p* < 0.05 were considered significant.

## Results

### CG-598 Is a HIF-1α Stabilizer Enhancing Barrier Proteins

CG-598 was discovered in the effort of CrystalGenomics, Inc. to investigate the selective HIF-PHD inhibitors. The generic structure of CG-598 is shown in **Figure 1A**, where X, Y, Z, and W are nitrogen, NO, CO, and R_2_ are hydrogen or alkyls, and E is NH or NH-alkyls (**Figure 1A**). The full structure of CG-598 and X-ray crystal structure bound in HIF-PHD1 protein would be disclosed soon. CG-598 efficiently inhibited the activity of HIF-PHD2, as the IC_50_ was 0.02 µM and it was more effective compared to previously developed HIF-PHD inhibitors (**Supplementary Figure 1A**). CG-598 is a pan HIF-PHD inhibitor because it efficiently inhibited both HIF-PHD1 (IC_50_ = 0.03 µM) and HIF-PHD3 (IC_50_ = 0.08 µM). When HeLa cells were treated with 10 µM CG-598, HIF-1α but not HIF-2α was stabilized similar to AKB-4924, although CoCl_2_ enhanced both (**Figure 1B** and **Supplementary Figure 1B**). Intestinal epithelial cells were not affected by CG-598 at steady state, and CG-598 induced ITF and CD73 expression under cellular stress by 3% DSS treatment (**Figure 1C** and **Supplementary Figure 1C**). DSS treatment damaged intercellular junctions including ZO-1, occludin, and E-cadherin (**Figures 1D–F**). CG-598 treatment maintained these junctional proteins similar to AKB-4924 treatment. Intestinal monolayer epithelium was established from Caco-2 cells in the upper well insert and cells were treated with 3% DSS to damage barrier integrity, CG-598 as well as AKB-4924 reduced the migration of Jurkat T-cells through the epithelial barrier (**Supplementary Figure 1D**). These results suggest that CG-598 treatment reinforces the barrier-associated molecules *via* HIF-1α stabilization to maintain barrier integrity under chemical insults similar to the effect of AKB-4924.

### CG-598 Acts as a Local HIF-1α Stabilizer in the Gut Without Systemic Effects

**Figure 2:**
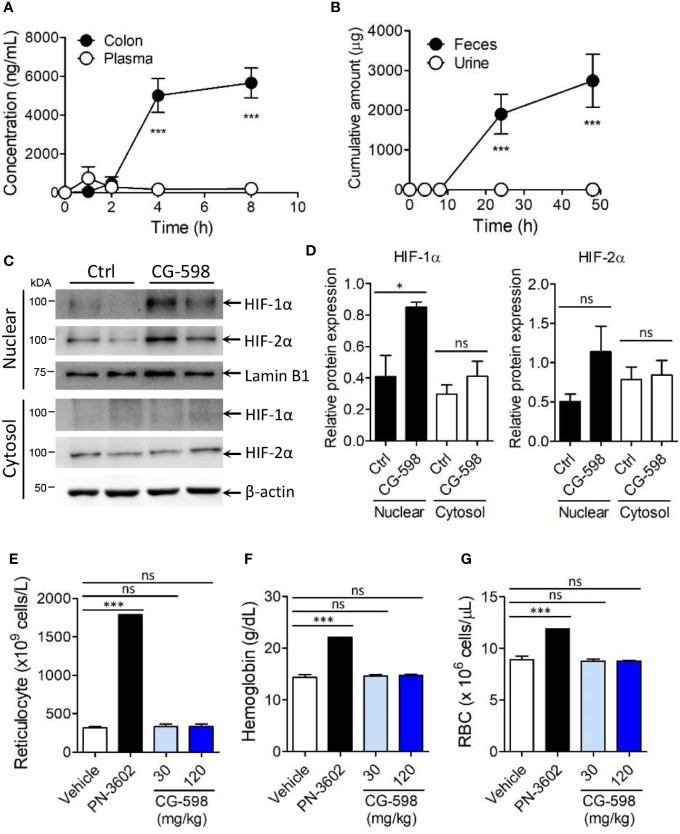
CG-598 is localized in gut tissue without systemic effects. **(A)** Mice were orally treated with 50 mg/kg CG-598 (n=3 per each time point). At each indicated time point, the concentration of CG-598 was quantified from plasma and colon tissues. **(B)** Rats were orally treated with 150 mg/kg CG-598 (n=3). Urine and feces were collected during 0–4 h, 4–8 h, 8–24 h, and 24–48 h and the amount of CG-598 was quantified and summarized cumulatively. **(C, D)** Mice were orally treated with 15 mg/kg of CG-598 once a day for 10 days (n=3 per group). **(C)** Tissue homogenates were analyzed to detect protein levels of cytosolic or nuclear HIFs in colon. β-actin and lamin B1 were used as cytosolic versus nuclear fraction controls, respectively. **(D)** Relative band intensities of HIF-1α and HIF-2α as compared to β-actin or lamin B1 were calculated. **(E–G)** Mice were orally treated with the indicated doses of CG-598 or 100 mg/kg PN-3602 once a day for 15 days (n=5 per group). Hematology parameters at day 15 were determined as the levels of **(E)** reticulocytes, **(F)** hemoglobin, and **(G)** red blood cells. Data are representative of three independent experiments. One-way ANOVA. Data are shown as mean ± SEM, ns, not significant, *p < 0.05, ***p < 0.001.

To optimize treatment of CG-598 for the *in vivo* study, mice were orally administered 50 mg/kg of CG-598 in various formulations and then its concentrations were analyzed in the intestine. CG-598 formulated in 1% CMC showed high and consistent distribution in the intestinal tissue after 4 h following administration (**Supplementary Figure 2A**). Dose-dependent absorption of CG-598 was high specifically in the intestine (**Supplementary Figure 2B**) but not in the plasma, suggesting that CG-598 can be localized in the gut without systemic absorption (**Figure 2A**). The majority of CG-598 was excreted through feces with little elimination through urine, also suggesting that it was not absorbed into the systemic compartment (**Figure 2B**). HIF-1α in the nuclear compartment was stabilized in the intestine of mice treated orally with CG-598 (**Figures 2C, D** and **Supplementary Figure 2G**). HIF-2α in the nuclear compartment was slightly increased following oral CG-598 treatment but the comparison to the level of HIF-2α in the control group was not significant. To confirm the local effect of CG-598 confined in the intestinal tissue, mice were orally fed with various doses of CG-598 once a day for 15 days. When body weight was monitored every four days following the first oral administration of CG-598, there were no significant decreases compared with vehicle groups (**Supplementary Figure 2C**). Hypoxia and HIF-1α stabilization can induce the generation of reticulocytes/red blood cells (RBCs) from bone marrow (31). At day 15, the number of reticulocytes/RBCs and the hemoglobin content in the CG-598 treated groups were not changed compared to the vehicle group, although oral treatment of 100 mg/kg PN-3602, a candidate of systemic distributed HIF-1α stabilizers for use of anemia therapy, significantly increased all parameters (**Figures 2E–G** and **Supplementary Figure 2D**). Treatment with CG-598 over 15 days at high doses did not show abnormal erythropoietin responses in hematology. These results suggest that oral administration of CG-598 could function as a local HIF-1α stabilizer in the gut without significant systemic effects.

### CG-598 Reduces the Severity of Intestinal Inflammation

**Figure 3:**
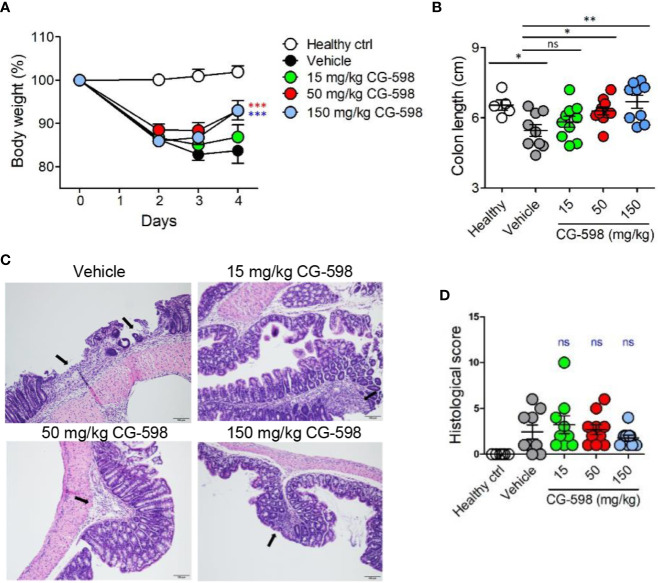
CG-598 mitigates DNBS-induced colitis in a dose-dependent manner. Mice were administered intra-rectally with 3% DNBS (n=9 or 10 per group). Three different doses of CG-598 or vehicle formulation were orally administered once a day. **(A)** Body weights. Two-way ANOVA. ***p < 0.001, compared to the vehicle group. **(B)** Colon lengths at day 4. One-way ANOVA. ns; not significant, *p < 0.05, **p < 0.01. **(C)** Representative colon histology from each group (×200 magnification). Black arrows mark severely inflamed regions. **(D)** Histological score. Data are representative of three independent experiments. One-way ANOVA. Data are shown as mean ± SEM, ns, not significant.

**Figure 4:**
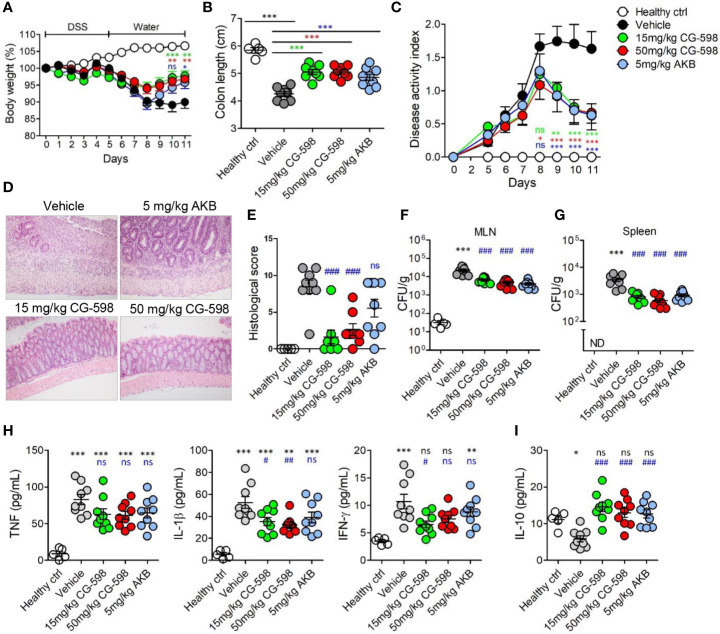
CG-598 reduces the severity of DSS-induced colitis. Mice were fed with 2.5% DSS in drinking water for five days and changed to normal water afterwards (n=9 per group). Mice were treated with CG-598 or AKB-4924 by oral gavage once every day. **(A)** Body weights. Two-way ANOVA. **(B)** Colon length at day 11. **(C)** Disease activity index. Two-way ANOVA. **(D)** Representative H&E stained colon tissues at day 11 (×200 magnification) and **(E)** Histological score. **(F)** Bacterial CFU counts in MLNs and **(G)** spleens isolated at day 11. **(H)** Levels of TNF-α, IL-1β, IFN-γ and **(I)** IL-10 were analyzed from colon tissue homogenates at day 11. Data are representative of three independent experiments. One-way ANOVA. Data are shown as mean ± SEM, **(A–C)** compared to vehicle group. **(E–I)** ns; not significant, *p < 0.05, **p < 0.01, ***p < 0.001 compared to healthy control group, #*p* < 0.05, ##*p* < 0.01, ###*p* < 0.001 compared to vehicle group.

To investigate whether local HIF-1α stabilization by CG-598 could protect against intestinal inflammatory insult, we used two animal models including DNBS-induced and DSS-induced colitis representing experimental murine models for Crohn’s disease and ulcerative colitis, respectively. When mice were orally treated with various doses of CG-598 following intra-rectal DNBS administration, the loss of body weight in CG-598 treated groups lessened and at day 4 showed a quick recovery and was significantly different from the vehicle group (**Figure 3A**). The lengths of colon were also recovered in the CG-598 treated groups in dose-dependent manner (**Figure 3B**). Colon tissues from mice treated with 150 mg/kg CG-598 showed reduced levels of crypt damage and ulceration (**Figures 3C, D** and **Supplementary Figure 2E**). However, since the histological grades of CG-598 treated was not that significant, we did further study using DSS-induced colitis. In DSS-induced colitis, CG-598 treatment relieved symptoms of colitis such as body weight loss, shortened colon length, and disease activity index (**Figures 4A–C**). After examination of colon tissues, all parameters including inflammation, crypt damage, and ulceration were significantly reduced in the CG-598 treated groups (**Figures 4D, E** and **Supplementary Figure 2F**). AKB-4924 treatment also reduced loss of body weight, disease activity index, and shortened colon length, but could not improve histological features of colitis (**Figure 4E**). Bacterial translocations were analyzed in the MLN and spleen at day 11. In healthy control mice, little or no culturable bacteria were counted in MLN or spleen, respectively (**Figures 4F, G**). However, bacteria counts were significantly increased in the MLN and spleen of the vehicle-treated group under DSS-induced colitis, suggesting that intestinal inflammation loosens gut barrier integrity to allow bacterial translocation into systemic tissues. CG-598 and AKB-4924 treatments significantly reduced bacterial translocation into MLN and spleen (**Figures 4F, G**). Consistent with this finding, the production of inflammatory cytokines such as IL-1β and IFN-γ was significantly reduced in colon tissues of the CG-598 treated group as compared to the vehicle treated colitis group (**Figure 4H**). The levels of TNF were slightly decreased in both CG-598 and AKB-4924 groups but it was not statistically meaningful. Production of the anti-inflammatory cytokine IL-10 decreased in the vehicle group as compared to the healthy control group. However, the amount of IL-10 was significantly increased in the CG-598 treated group compared to that of the vehicle group (**Figure 4I**).

### CG-598 Fortifies Gut Barrier Integrity and Immune Regulation

**Figure 5:**
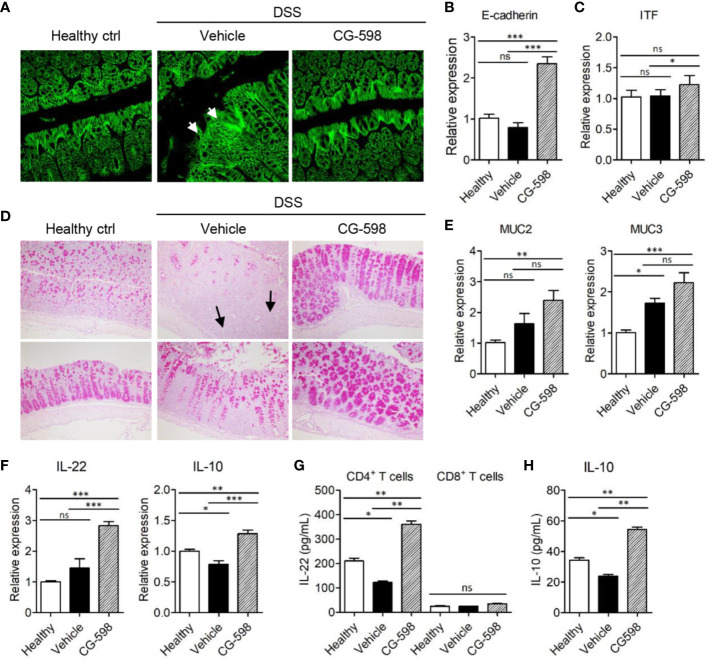
CG-598 enhances barrier function and immune regulation during colitis. Mice were fed with 2.5% DSS in drinking water for five days and followed by normal water through the end of the experiments (n=9 per group). Mice were treated with 15 mg/kg CG-598 by oral gavage once every day. Colon tissues were analyzed at day 11. **(A)** E-cadherin staining (×400 magnification). **(B)** Relative mRNA expression of *E-cadherin* and **(C)** *itf* to *gapdh*. **(D)** Periodic acid–Schiff staining of colon tissues. (×200 magnification) **(E)** Relative mRNA expression of *muc2* and *muc3* and **(F)** IL-22 and IL-10 in colon tissue. **(G, H)** CD4^+^ T-cells or CD8^+^ T-cells were sorted from lamina propria of colon tissues at day 11 and stimulated with anti-CD3/CD28 antibodies overnight. The levels of IL-22 from T-cells and IL-10 from CD4^+^ T-cells were analyzed from culture supernatants. One-way ANOVA. Data are shown as mean ± SEM, ns, not significant, *p < 0.05, **p < ***p < 0.001.

To investigate the *in vivo* effect of CG-598 on gut barrier function, the cell adhesion molecule E-cadherin, which is important for the formation of adherens junctions, was analyzed. DSS treatment induced focal damage of the E-cadherin network in the colon epithelium of vehicle-treated mice (**Figure 5A**). Mice treated with CG-598 under DSS-induced colitis showed well-organized structures of epithelial adherens junctions and higher expression levels of *E-cadherin* compared to the vehicle group (**Figures 5A, B**). Expression of tight junction proteins occludin and ZO-1 increased as well (**Supplementary Figure 2G**). In addition, the expression of *itf* was significantly increased in colons from mice treated with CG-598 (**Figure 5C**). Mucus production from goblet cells was abundant in the colon of CG-598 treated mice as compared to healthy or vehicle-treated tissues (**Figure 5D**). In the vehicle-treated colon, few goblet cells existed in the inflamed areas of crypt loss (upper panel) although goblet cells could produce mucin in the less inflamed regions (lower panel). Expression levels of *muc2* and *muc3* were significantly increased in colons treated with CG-598 (**Figure 5E**). The expression of IL-22 and IL‑10 were both significantly increased in colons treated with CG-598, which are critical for barrier maintenance and immune regulation, respectively (**Figure 5F**). To investigate which cell type produces these cytokines, immune cells were isolated from the lamina propria of colon after CG-598 treatment under DSS colitis and analyzed for production of cytokines. CG-598 treatment enhanced secretions of IL-22 and IL‑10 from CD4^+^ T-cells (**Figures 5G, H**). CD8^+^ T-cells produced little IL-22 (**Figure 5G**) while IL-10 was not detected (data not shown) regardless of CG-598 treatment. Foxp3^+^ Treg populations were increased in the lamina propria of inflamed colon under DSS colitis, and although CG-598 treatment slightly increased Treg populations, it was not significantly different compared to that of the vehicle-treated group (**Supplementary Figures 3A–C**). These results suggest that CG-598 treatment could endow resistance to intestinal immune insults by enhancing both the physical barrier integrity of the epithelium and the immune regulatory machinery through CD4^+^ T-cells cytokine production.

### The Therapeutic Effect of CG-598 Is Comparable or Better Than Other IBD Therapeutics

**Figure 6:**
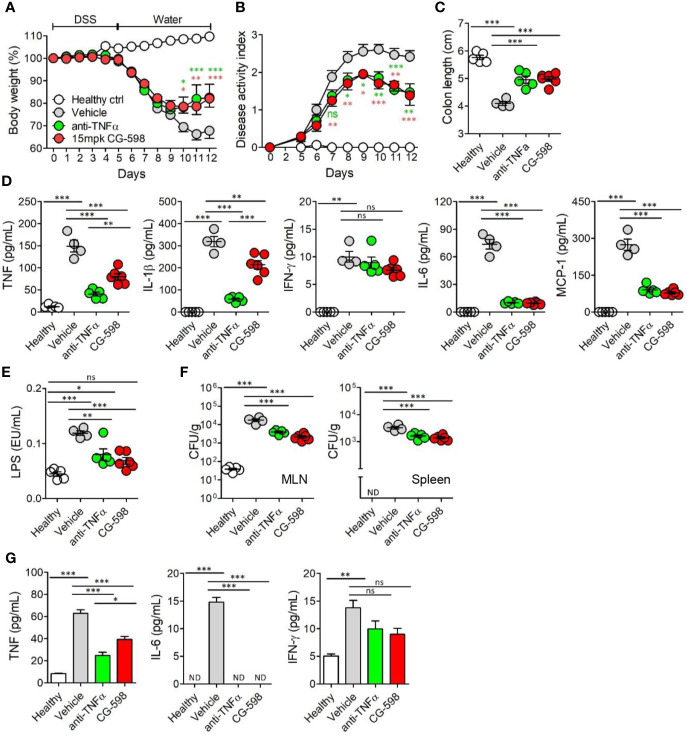
Oral CG-598 treatment has a comparable protective effect to TNF blocker against colitis. Mice were fed with 2.5% DSS in drinking water for five days followed by normal water afterwards (n=8 per group). Mice were treated with 15 mg/kg CG-598 by oral gavage once every day. Animals were treated with anti-mouse TNF-α antibody (0.5 mg per mouse) intraperitoneally at days 4, 6, 8, and 10. **(A)** Body weight and **(B)** disease activity index following DSS treatment. Two-way ANOVA. **(C)** Colon length at day 12. **(D)** Cytokines from colon tissue homogenates. **(E)** Serum lipopolysaccharide concentrations. **(F)** Bacterial CFUs cultured from MLN (left) and spleen (right). **(G)** Serum inflammatory cytokines. Data are representative of three independent experiments. One-way ANOVA. Data are shown as mean ± SEM, ns, not significant, *p < 0.05, **p < 0.01, ***p < 0.001, compared to the vehicle group.

To compare the efficacy of current regimens for IBD therapeutics, oral CG-598 treatment was investigated in comparison with an anti-TNF blocker in DSS-induced colitis. TNF-α is a critical pro-inflammatory cytokine in the pathogenesis of IBD which also increases gut barrier permeability (29). We investigated whether oral CG-598 treatment could ameliorate colitis comparable to an anti-TNF blocker by evaluation of body weight changes, disease activity index, and shortened colon lengths (**Figures 6A–C**). As expected, the levels of TNF and IL-1β from colon tissues of mice treated with the anti-TNF blocker were significantly reduced compared to that of the vehicle or CG-598 treated groups (**Figure 6D**). CG-598 treatment also significantly decreased the production of pro-inflammatory cytokines such as TNF, IL-1β, IL-6 and MCP-1 but not IFN-γ. When gut barrier functions were assessed, oral CG-598 treatment reduced the level of serum LPS and bacterial translocation into MLN or spleen, which were comparable to the actions of the TNF-blocker suggesting enhancement of gut barrier integrity (**Figures 6E, F**). Consistent with these results, the levels of pro-inflammatory cytokines in the serum, TNF and IL-6, were significantly reduced (**Figure 6G**). The levels of serum IFN-γ in the oral CG-598 and TNF-blocker groups were slightly reduced but not statistically different with that of the vehicle group. When compared to treatment with tofacitinib (32), a JAK inhibitor, oral CG-598 treatment had similar effects to tofacitinib in alleviating body weight loss (**Supplementary Figure 4A**). In the aspect of disease activity index, colon length and pro-inflammatory cytokines, the anti-colitic effect of CG-598 treated group was comparable with that of tofacitinib treated group (**Supplementary Figures 4B–D**). Tofacitinib and CG-598 were also treated in combination to investigate their synergistic effect, however, did not show significant difference when treated individually (**Supplementary Figures 4A–D**). Taken together, oral treatment of CG-598 has beneficial effects as compared with direct and systemic anti-inflammatory regimens such as TNF-blockers or JAK inhibitors because CG-598 exerts selective protective effects against intestinal inflammation *via* reinforcement of gut barrier integrity and immune regulation. However, direct comparison for efficacy with commercial drugs has limitations since optimal doses, formulation and pharmacokinetics may differ between the therapeutics.

## Discussion

The intestinal mucosa has multi-layer barriers to maintain immunological homeostasis (3). Most of the outer layer is a microbial barrier that consists of large numbers of commensal microbiome to compete against pathogens. The next layer is a mucous barrier with antimicrobial peptides which are produced by goblet cells and Paneth cells. The third layer is a monolayer epithelium tightly interconnected with tight and adherens junction including claudins, occludin, ZO-1 and E-cadherin. The final barrier must be an immunological surveillance system to detect and clear microbial invaders. These multi-layered gut barriers sustain sterile conditions in most of the organs within our body. In the low oxygen microenvironment of the gut, fermented short-chain fatty acid metabolites from dietary fibers help epithelial cells to fortify barrier functions in the villous epithelium. Intestinal epithelial cells *via* hypoxia-induced adaptation increase barrier protective proteins such as ITF, CD73, CD55, mucin-3 and MDR1 (10). The differential roles of HIF-1α vs HIF-2α in the intestinal epithelial cells have been well-studied in experimental colitis models. Epithelial HIF-1α has a protective role by preserving epithelium barrier function (16). Previous studies show that PHD inhibitors exert protective effects during experimental colitis by fortifying the gut barrier. The pan-PHD inhibitor, AKB-4924 preferentially stabilizes HIF-1α rather than HIF-2α (22). PHD inhibitors ameliorate intestinal inflammation in murine experimental TNBS colitis by increasing barrier-related gene expression and are currently under investigation as IBD therapeutics in clinical trials. CG-598 could be proposed as one of the therapeutic options for IBD by targeting gut barriers but not for anti-inflammation.

HIF signaling is associated with many human cancers, including colorectal carcinoma. Expression of HIF-2α induces colorectal cancer (33, 34), but HIF-1α does not (35). Our data show that CG-598 stabilizes HIF-1α in the colon. The potential adverse effects of PHD inhibitors are associated with its systemic exposure followed by increased HIF target gene expression in extra-intestinal organs. Systemic stabilization of HIF-1α leads to erythropoiesis and angiogenesis and may lead to polycythemia (36, 37). To reduce systemic off-target effects, maintenance of distribution and stabilization activity of HIF-1α locally to the gut is critical for development of IBD therapeutics (10). Oral treatment with the pan-hydroxylase inhibitor DMOG was not effective in murine colitis and development of a different formulation strategy is needed for oral delivery (38). Oral AKB-4924 treatment mainly exerted local HIF stabilization in the gut but not in the kidney and heart (23). In addition, oral AKB-4924 treatment did not affect erythropoietin and hematocrit, suggesting that their effects were kept local. Furthermore, AKB-4924 diminished bacterial translocation and restored goblet cells, which limited physical interaction with luminal bacteria (39). Our data show that oral delivery of CG-598 led to local distribution to the gut even in high doses. Little distribution of CG-598 in the plasma reduces off-target effects such as alteration of hematology parameters in the systemic compartment and suggests selective local therapeutics for gut inflammation.

HIFs are involved with pro-inflammatory but also anti-inflammatory adaptive responses, depending on environmental conditions and cell types. In addition, HIFs can control metabolism and function of immune cells. For energy production, M1 macrophages mainly utilize glycolysis whereas M2 macrophages rely on oxidative phosphorylation (40). HIF-1α specifically controls the glycolytic pathway in macrophages and promotes M1 polarization because HIF-1α deficiency in macrophages fails to induce survival, migration and bacterial killing (41). Macrophages infected by fungi show an inverse relationship between IL-10 and HIF-1α (42). However, activation of HIF-1α in macrophages within the periapical mucosa negatively regulates inflammation *via* decreasing the ratio of M1/M2 (43). HIF-1α in colonic myeloid immune cells is critical for resolution of inflammatory injuries (44). Depletion of microenvironmental oxygen in the gut epithelium by trans-migrating neutrophils stabilize HIFs on epithelial cells which confers protection from colitis (39). In dendritic cells, HIF-1α induces type I interferon and IL-10 (45). HIF-1α deficiency specifically in dendritic cells is prone to induce intestinal inflammation because Treg development is impaired with reduced CCR9 expression and retinoic acid utilization (45). Therefore, the roles of HIFs in innate immune cells following CG-598 treatment for reduced gut inflammation require further investigation.

In T-cells HIF-1α acts as a key metabolic sensor which reduces Treg development and Th1 effector function (46) but enhances Th17 development (47). In fact, the effect of HIF-1α on Tregs is rather conflicting. Some studies report that HIF-1α negatively regulates Foxp3 expression in Tregs (47, 48). In some other reports, HIF-1α induces Foxp3 and drives the generation and function of Tregs indicating that HIF-1α-deficient Tregs fail to control T-cell-mediated colitis (49, 50). Consistent with this finding, HIF-1α deficiency particularly in T-cells exacerbates colonic inflammation in murine DSS-induced colitis (51). Under hypoxia, even Th17 can produce IL-10 suggesting their immune regulatory function (52). In CD4^+^ T-cells, HIF-1α induces the production of IL-22 which is critical for barrier function in the gut mucosa *via* increasing proliferation of epithelial cells and expression of mucins and antimicrobial peptides (53, 54). HIF-1α drives IL-10 expression in B-cells in controlling autoimmune inflammation such as arthritis and experimental autoimmune encephalomyelitis (55). Hypoxia reduces intestinal inflammation through down-regulation of NLRP3/mTOR and autophagy activation (56). CG-598 treatment did not directly affect thymic-derived or peripheral Treg cell populations in the intestinal lamina propria. However, CG-598 treatment induced the production of IL-10 and IL-22 from lamina propria CD4^+^ T-cells suggesting that strengthening HIF-1α induction in T-cells at sites of inflammation might be a therapeutic strategy for IBD management.

Treatment with TNF-α blockers offers an efficient way to control pathologic inflammation in IBD as well as various other chronic inflammatory diseases. Although anti-TNF therapy agents such as infliximab and adalimumab are broadly used in patients with IBD, the outcome is not always successful and adverse effects such as infections frequently occur. TNFR1 is associated in intestinal epithelial cell proliferation (57). Some reports suggest that murine colitis worsened after anti-TNF blockade (58, 59). In a T-cell mediated colitis model, TNFR2 is required for stabilization of Tregs (60). In addition, the clinical use of TNF blockers is closely associated with an enhanced risk for a variety of serious microbial infections, because TNF-α plays a critical function in mediating initial protective immune responses against pathogenic microbes. In fact, TNF-α is critical to defend against bacterial pathogens infection including *Mycobacterium tuberculosis* and fungal infections such as *Candida albicans*, *Aspergillus fumigatus* and *Cryptococcus neoformans* (61). Therefore, prior to the administration of TNF-α blockers, a variety of medical recommendations recommend pre-screening for important infections, including tuberculosis and hepatitis (62).

In this study we showed the positive effects of CG-598 in gut barrier protectivity, however some limitations remain to be unveiled. CG-598 required at least a dose of 15 mg/kg to show protective effects on murine colitis model, which was higher than 5 mg/kg AKB-4924. This may be due to the absorption character of the drug. Nonetheless, the effects of CG-598 in ameliorating colitis were dramatic. Similar with CG-598, the anti-colitic 5-aminosalicylic acid based drugs such as sulfasalazine and olsalazine which are known for non-absorbable IBD therapeutics, requires a relatively high dose of 30-100 mg/kg in the murine DSS-induced colitis model (63). However, local distribution of non-absorbable CG-598 did not affect extra-intestinal organs and was directly excreted through the feces anticipating high safety of the drug (**Figure 2**). Also, increased levels of IL-22 were detected in the colon tissue and colonic CD4^+^ T-cells, however we could not detect IL-17 level changes in CD4^+^ T-cells (data not shown). Although IL-22 is closely related to IL-17, there are cell subsets such as Th22 cells that can secrete IL-22 independently of IL-17 (64, 65). The source of IL-22 production induced by CG-598 needs to be revealed in future studies.

Taken together, these results support CG-598 as a novel candidate for IBD therapeutics because it is optimized for local stabilization of HIF-1α in the gut. The protective effect of CG-598 is associated with increased epithelial barrier integrity together with immune regulation *via* IL-22 and IL-10. CG-598 has no expected off-target and adverse effects such as abnormal hematology or enhanced incidence of infection. Our findings demonstrate CG-598 as an effective and safe candidate for IBD therapeutics and provide critical insight into therapeutic options for inflammatory mucosal disease.

## Data Availability Statement

The datasets presented in this study can be found in online repositories. The names of the repository/repositories and accession number(s) can be found in the article/**Supplementary Material**.

## Ethics Statement

The animal study was reviewed and approved by Institutional Animal Care and Use Committee of Crystal Genomics (CG-IACUC-19017), Institutional Animal Care and Use Committee of Ajou University (IACUC No. 2017-0021).

## Author Contributions

Y-IK, Y-RH, and S-YC conceived and designed experiments, Y-IK, E-JY, Y-DK, AL, JC, HH, S-RK performed experiments and analysis, JMC provided critical materials, Y-IK, H-JK, J-HC, YH, and S-YC wrote the manuscript and provided creative input. All authors contributed to the article and approved the submitted version.

## Funding

This work was supported by Ajou University Research Fund (2019), Republic of Korea and the National Research Foundation of Korea (NRF), funded by the Ministry of Science, ICT and future Planning (grant numbers NRF-2019R1I1A1A01057559, NRF-2020R1A2B5B01001690).

## Conflict of Interest

Y-DK, AL, JC, HH, JMC, and YH were employed by the company CrystalGenomics, Inc.

The remaining authors declare that the research was conducted in the absence of any commercial or financial relationships that could be construed as a potential conflict of interest.
