# Supplementary material for: Local Stabilization of Hypoxia-Inducible Factor-1α Controls Intestinal Inflammation via Enhanced Gut Barrier Function and Immune Regulation
Source: Front Immunol. 2021 Jan 14;11:609689. doi: 10.3389/fimmu.2020.609689 (PMC7840603; doi:10.3389/fimmu.2020.609689)
Supplement: Supplementary file 1 [file DataSheet_1.pdf]

A

HIF-PH2 inhibitors	IC_{50} (µM)
AKB-4924 (IBD candidate)	6.45
FG-4592 (Anemia candidate)	3.35
CG-598	0.02

B

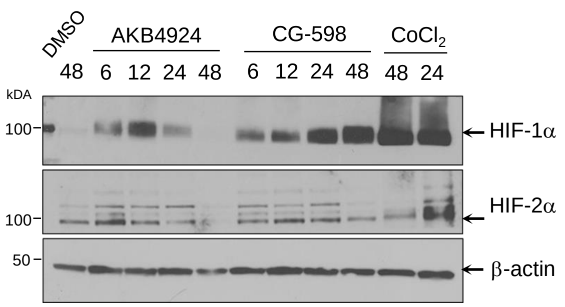

C

D

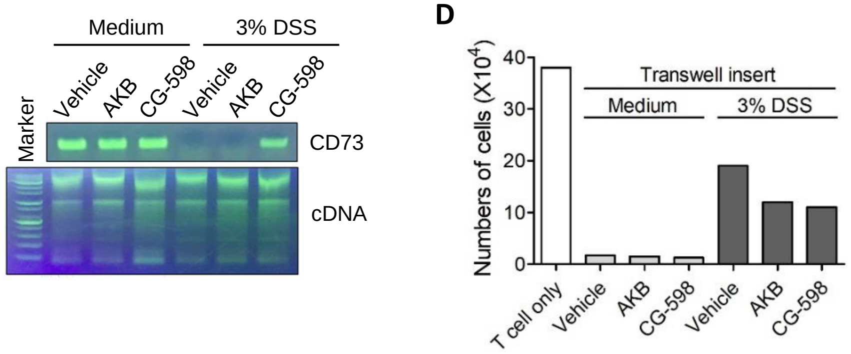

Supplementary Figure 1. CG-598 enhances CD73 expression and reduces T-cell migration trough in intestinal epithelial cells. (A) IC_{50} values for inhibition of HIF-PH2 enzyme. Results of HIF-PH2 activity assays for tested compounds using FP methods. Assay of each sample well was performed in duplicate and the assay was repeated three times independently with similar results. (B) HeLa cells were treated with 10 µM AKB4924, CG-598, or CoCl2 for 6, 12, 24, 48 h. Whole cell extracts from each treatment group were analyzed to detect HIF-1α and HIF-1β. (C) HCT116 cells were pre-treated with 10 µM AKB4924 or 10 µM CG-598 for 2 h and further incubated 3% DSS for 24 h. After incubation, cells were harvested for total RNA extraction followed by PCR. The mRNA level of CD73 and total RNA were detected by 1% agarose gel. (D) For T-cell migration assays, Caco-2 cell monolayers, grown in 24-transwell plates, were treated with AKB4924 or CG-598 for 1 h and cells were further incubated with 3% DSS for 72 h. Jurkat T-cells were then added to each plate. After incubation for 24 h, the number of Jurkat T-cells that passed through the transwell chamber was counted. All treatments were performed in triplicate and each assay was repeated two times independently with similar results.

Supplementary Figure 2

A

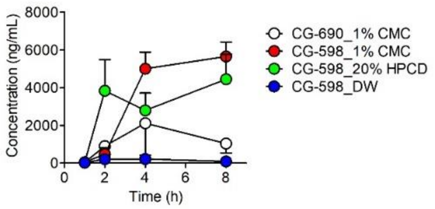

B

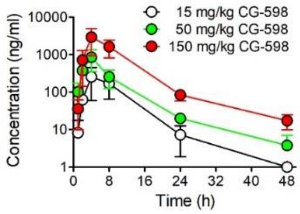

C

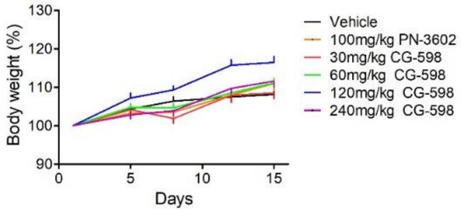

D

	RBC ($X10^6$ cells/μl)	Hemoglobin (g/dl)	Reticulocyte ($x10^9$ cells/L)	Reticulocyte (%)
Vehicle	8.91±0.804	14.4±1.125	317.66±35.351	3.59±0.493
PN-3602 100mg/kg	≥11.89	≥22.1	≥1788.1	≥15.04
CG-598 30mg/kg	8.79±0.399	14.62±0.622	332.66±74.120	3.78±0.822
CG-598 60mg/kg	8.87±0.246	14.8±0.469	339.82±60.796	3.83±0.692
CG-598 120mg/kg	8.77±0.170	14.72±0.487	338.38±65.832	3.86±0.761
CG-598 240mg/kg	9.482±0.108	16.08±0.545	340.32±45.665	3.59±0.473

(Mean±SEM)

E

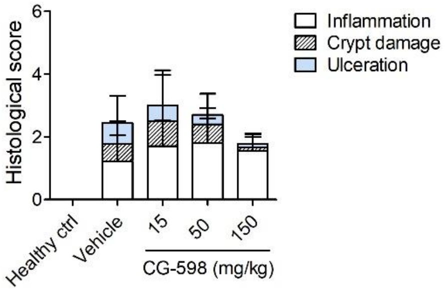

F

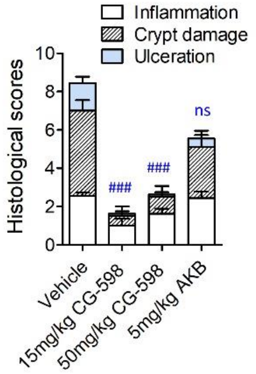

G

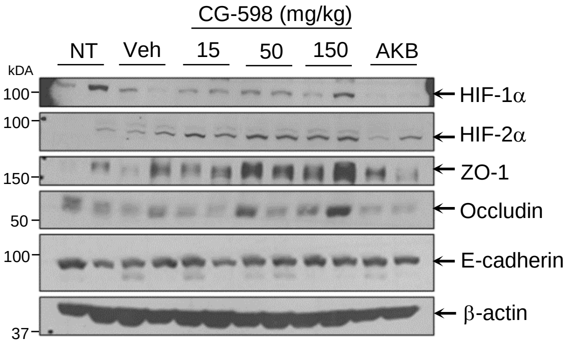

Supplementary Figure 2. Assessment of gut distribution and hematologic effects of CG-598.

(A) Mice were orally administered 50 mg/kg CG-598 or CG-690, the other candidate of HIF-PH inhibitor (n=3). Various compositions of vehicle media were tested for efficient delivery of drug. The concentrations of CG-598 from intestinal tissue homogenates were determined at each indicated time point. (B) Mice were treated with three oral doses of CG-598 resuspended in 1% CMC (n=4). The concentrations of CG-598 from intestinal tissue homogenates were determined at each indicated time point. (C) Mice were orally administered the indicated doses of CG-598 or PN-3602, a candidate for anemia therapeutics, once a day for 15 days (n=5). Body weights of mice were monitored every four days. (D) RBC hematology following 15 days treatment orally once a day. (E) Cumulative histologic scores including inflammation, crypt damage, and ulceration in the DNBS-induced colitis (related to Figure 3D) and (F) DSS-induced colitis (related to Figure 4E). (G) Mice were fed with 2.5% DSS in drinking water for five days and changed to normal water afterwards (n=2 per group). Mice were treated with CG-598 (15/50/150 mg/kg) or AKB4924 (5 mg/kg) by oral gavage once every day. At day 11, colon tissue homogenates were analyzed to detect protein levels HIFs, ZO-1, occludin and E-cadherin.

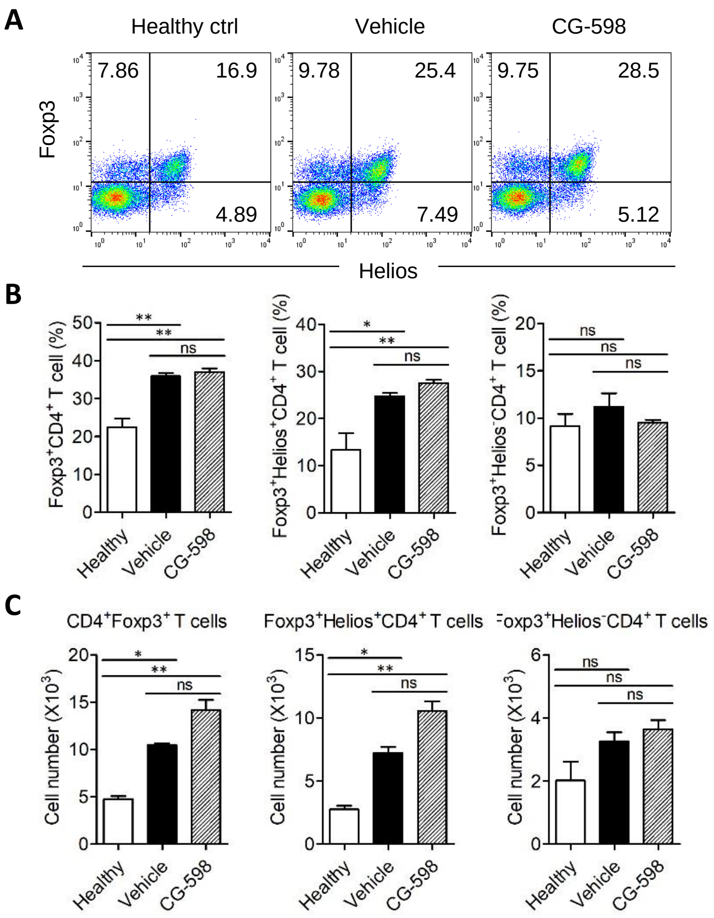

Supplementary Figure 3. Analysis of thymic versus peripheral Tregs following oral CG-598 treatment in the lamina propria of colon under DSS-induced colitis. Mice were fed with 2.5% DSS in drinking water for five days and followed by normal drinking water afterwards. Mice were treated with 15 mg/kg CG-598 by oral gavage once every day (n=3 per group). At day 11, $CD4^+$ T-cells were isolated from the lamina propria of colon and analyzed for expression of Foxp3 and Helios. (A) Representative flow cytometry histogram of colon Tregs. (B) Percentages of the $Foxp3^+$ population for each Treg subset including thymic ($Foxp3^+Helios^+$) and peripheral ($Foxp3^+Helios^-$) Tregs among $CD4^+$ T-cells. (C) Absolute cell number of Tregs. One-way ANOVA. ns: not significant, *$p<0.05$, **$p<0.01$.

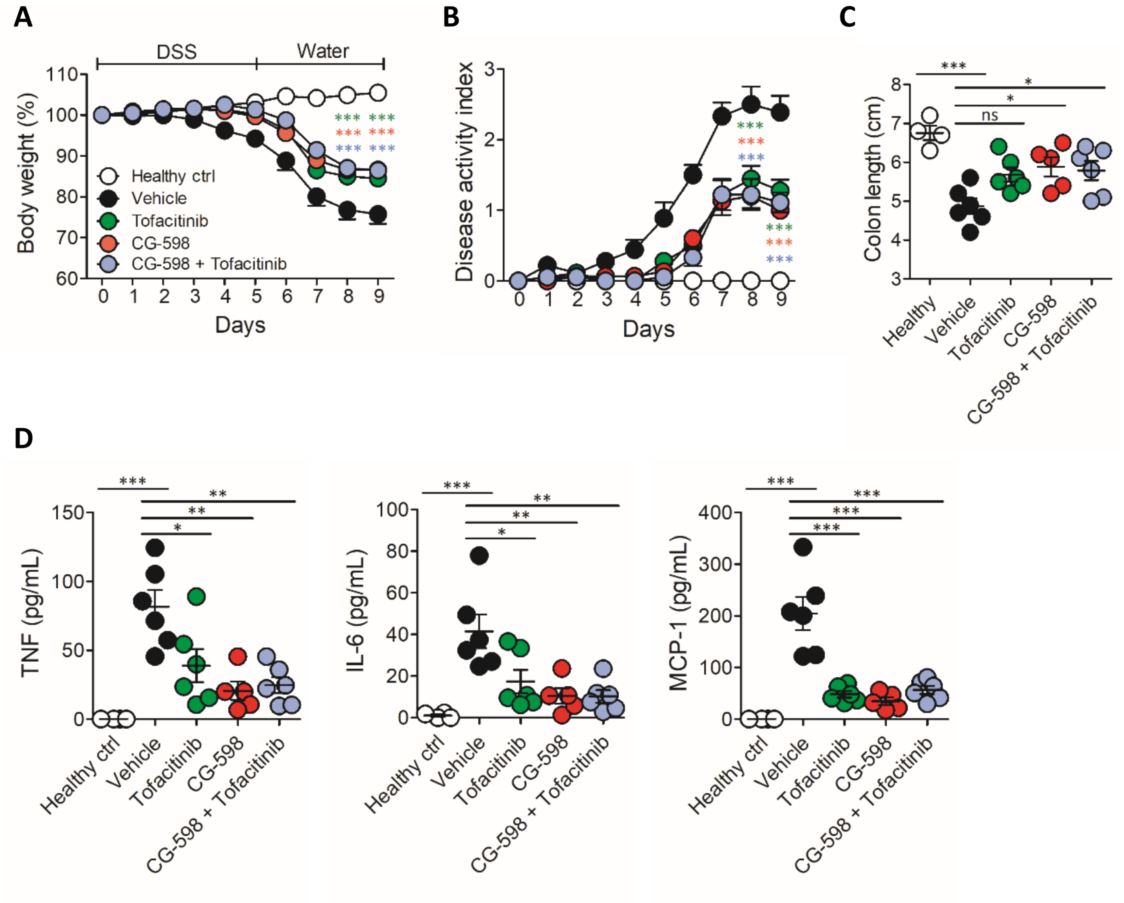

Supplementary Figure 4. Effect of CG-598 compared with tofacitinib in DSS-induced colitis. Mice were fed with 2.5% DSS in drinking water for five days followed by drinking water through the end of the experiment (n=6 per group). Mice were treated with 15 mg/kg CG-598 or 30 mg/kg tofacitinib by oral gavage once every day. (A) Body weight and (B) Disease activity index following DSS treatment. (C) Colon length at day 9. (D) Cytokines from colon tissue homogenates. One-way ANOVA. ns: not significant, *$p<0.05$, **p<0.01, ***p<0.001, compared to vehicle group.

Figure 5B

CT value	E-cadherin	GAPDH
Control 1	26.89552	20.92582
Control 2	26.93275	20.76834
Control 3	27.16371	21.39131
Control 4	27.43556	21.15337
Control 5	26.47294	21.22183
Control 6	26.87642	20.97088
Vehicle 1	27.316	20.49914
Vehicle 2	27.03803	20.56634
Vehicle 3	26.06874	20.91327
Vehicle 4	25.9715	20.79469
Vehicle 5	26.85239	19.49039
Vehicle 6	26.63154	19.75019
CG 1	24.98121	20.65367
CG 2	24.84589	20.37766
CG 3	24.7295	20.74452
CG 4	24.79625	20.64667
CG 5	25.50682	19.24098
CG 6	24.77723	19.87876

Figure 5C, F

CT value	IL-22	ITF	GAPDH
Control 1	31.07687	18.26261	20.9332
Control 2	30.89129	18.19223	20.47389
Control 3	30.94457	18.93028	20.76508
Control 4	30.73747	18.88767	20.86839
Control 5	31.04768	18.71993	
Control 6	31.00052	19.01255	
Vehicle 1	29.057	17.7887	20.32671
Vehicle 2	28.98937	17.67456	20.19308
Vehicle 3	30.32467	17.7277	19.15499
Vehicle 4	30.1562	17.83411	19.23316
Vehicle 5	30.53607	18.29251	20.67116
Vehicle 6	29.9906	18.25486	20.72875
CG 1	28.65343	18.07447	20.82958
CG 2	28.889	17.49867	20.68835
CG 3	28.83602	17.52189	20.38857
CG 4	28.40497	17.6031	20.64426
CG 5	28.91822	17.98535	19.07424
CG 6	28.94828	17.80934	18.84052

Figure 5E

CT value	MUC2	MUC3	GAPDH
Control 1	22.14519	21.9731	21.94005
Control 2	22.42817	22.07063	21.79252
Control 3	22.13031	22.45987	21.99211
Control 4	22.47602	22.38256	21.88435
Control 5	22.97055	22.07146	22.01967
Control 6	22.3414	21.93052	22.07758
Vehicle 1	20.34395	19.00018	20.32943
Vehicle 2	20.07287	19.80774	20.44467
Vehicle 3	19.15178	19.80328	19.12868
Vehicle 4	19.04501	19.63028	19.38806
Vehicle 5	20.79965	19.58429	20.79708
Vehicle 6	20.66042	19.40264	20.57691
CG 1	18.99059	19.42388	20.87402
CG 2	18.78204	19.33606	20.78655
CG 3	19.92596	19.14211	20.44149
CG 4	19.60381	18.97381	20.70068
CG 5	19.74053	19.23822	19.19869
CG 6	19.51825	19.36258	18.96017

Figure 5H

CT value	IL-10	GAPDH
Control 1	35.74482	20.79699
Control 2	35.7414	20.35106
Control 3	35.79026	20.93805
Control 4	Undetermined	20.87875
Control 5	35.78737	20.96749
Control 6	35.9786	20.72176
Vehicle 1	35.91122	20.27353
Vehicle 2	36.01833	20.21009
Vehicle 3	35.92091	20.21006
Vehicle 4	35.94637	20.24221
Vehicle 5	35.55281	20.67904
Vehicle 6	35.43143	20.81218
CG 1	34.70583	20.45695
CG 2	34.96812	20.19351
CG 3	34.70749	20.34944
CG 4	34.54656	20.38926
CG 5	34.92702	19.53917
CG 6	34.88707	19.72522

Supplementary Table 1. RT-PCR gene expression data sets of main Figure 5.
